# Recurrent Haemoptysis From Retained Endobronchial Sutures Successfully Managed Using Flexible Endobronchial Scissors

**DOI:** 10.1002/rcr2.70613

**Published:** 2026-05-27

**Authors:** Talal AlSaeed, Derar AlShehab, Abdullah Alelewah, Hassan Jamaluddin, Essa AlGunaim, Adel Ayed, Sulaiman Khadadah

**Affiliations:** ^1^ Thoracic and Foregut Surgery Unit, Department of Thoracic Surgery Chest Disease Hospital Sabah Health Region Kuwait; ^2^ College of Medicine and Health Sciences, Abdullah Al Salem University Sabah Health Region Kuwait; ^3^ Interventional Pulmonary Unit, Department of Thoracic Surgery Chest Diseases Hospital Sabah Health Region Kuwait

**Keywords:** bronchoscopy, endobronchial sutures, foreign body removal, haemoptysis, lobectomy complications

## Abstract

Haemoptysis can be a challenging diagnostic dilemma. The most common causes include acute respiratory infection, cancer, bronchiectasis and chronic obstructive pulmonary disease. However, in 20% of cases no clear cause can be found. We present here a rare case of a 54‐year‐old male who previously had a lobectomy for lung cancer and presented with chronic low‐grade haemoptysis caused by endobronchial polypropylene sutures. We detail their successful removal using a novel technique with flexible endobronchial scissors and flexible fibreoptic bronchoscopy under light sedation. This case highlights a rare cause of haemoptysis that can be easily missed without bronchoscopy. It also shows that flexible endoscopic scissors can be safely used in the airway with a flexible bronchoscope to remove a foreign body that is implanted in the airway, and reviews surgical techniques to prevent sutures in the airway.

## Graphical abstract

We report a rare case of recurrent haemoptysis caused by retained endobronchial polypropylene sutures 14 years after lobectomy. The sutures were safely identified and removed using flexible endobronchial scissors via fibreoptic bronchoscopy, resulting in complete resolution of bleeding. This case highlights a minimally invasive and effective approach for managing suture‐related airway complications.

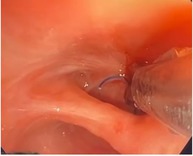

## 1 Introduction

Haemoptysis can be a challenging diagnostic dilemma. It is defined as the expectoration of blood from the lower respiratory tract [1, 2]. The most common causes include acute respiratory infection, cancer, bronchiectasis and chronic obstructive pulmonary disease [1, 2]. However, in 20% of cases no clear cause can be found [1]. In the postoperative setting, late‐onset haemoptysis following lobectomy is rare and may be attributed to multiple causes including bronchiectasis, local infection or foreign‐body reaction to surgical materials [3]. Non‐absorbable sutures, particularly polypropylene, are durable and biocompatible, yet when exposed to the airway lumen they can act as chronic irritants, inciting granulation, chronic cough and intermittent bleeding [4]. Traditionally, cases like this were managed via rigid bronchoscopy or surgical intervention; however, with advances in flexible interventional bronchoscopy we can achieve similar results under conscious sedation. We present a rare case of chronic low‐grade haemoptysis caused by endobronchial polypropylene sutures and their successful removal using flexible endobronchial scissors via flexible bronchoscopy.

## 2 Case Report

**FIGURE 1:**
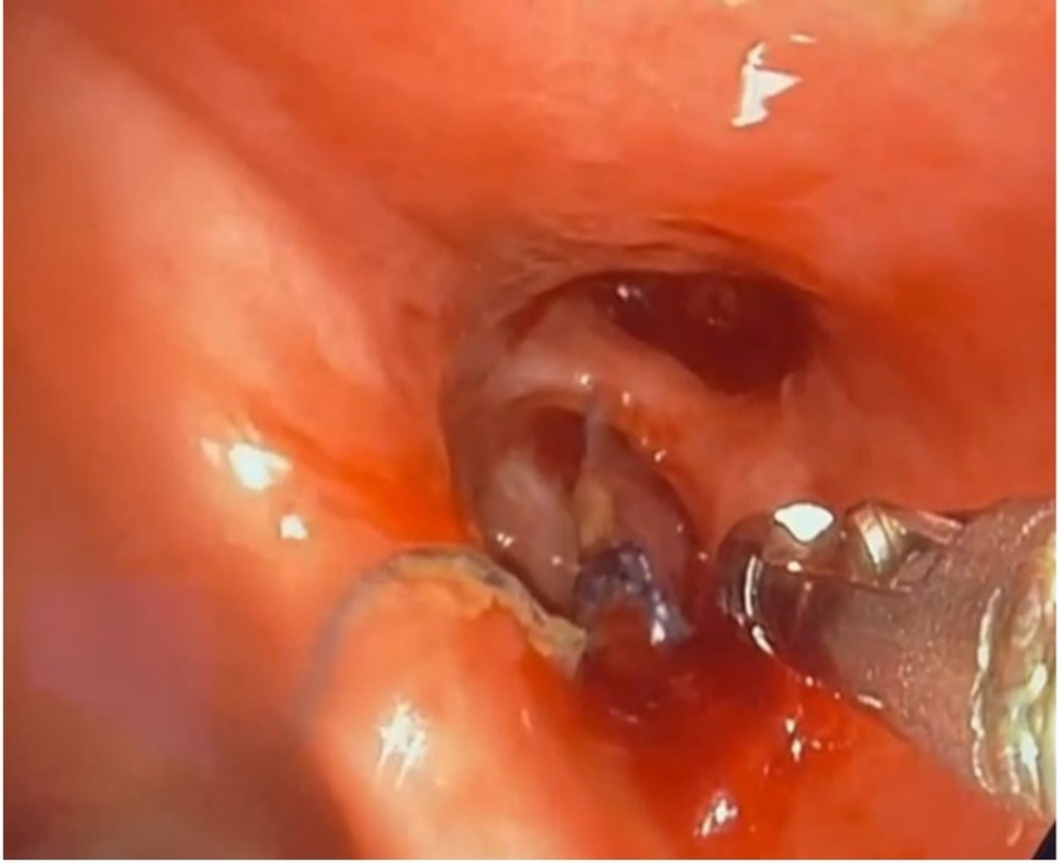
Endobronchial sutures embedded in the granulation tissue.

**FIGURE 2:**
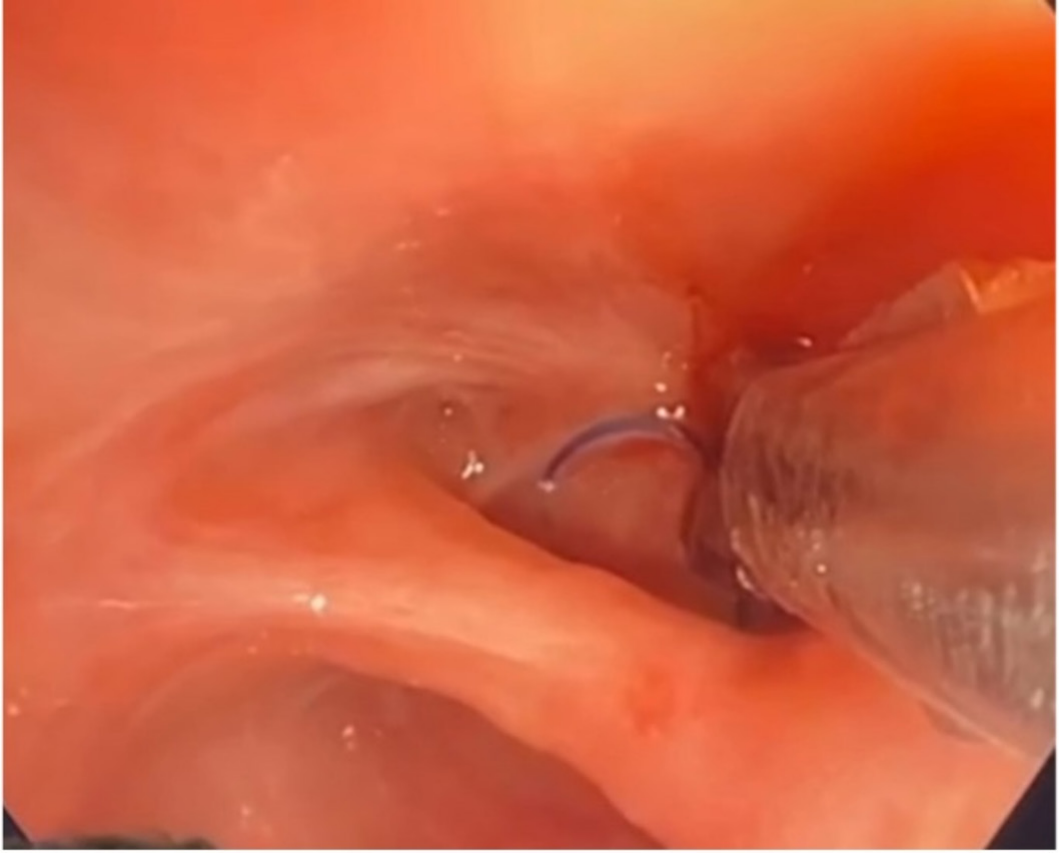
Endoscopic scissors with the endobronchial suture between its jaws.

**FIGURE 3:**
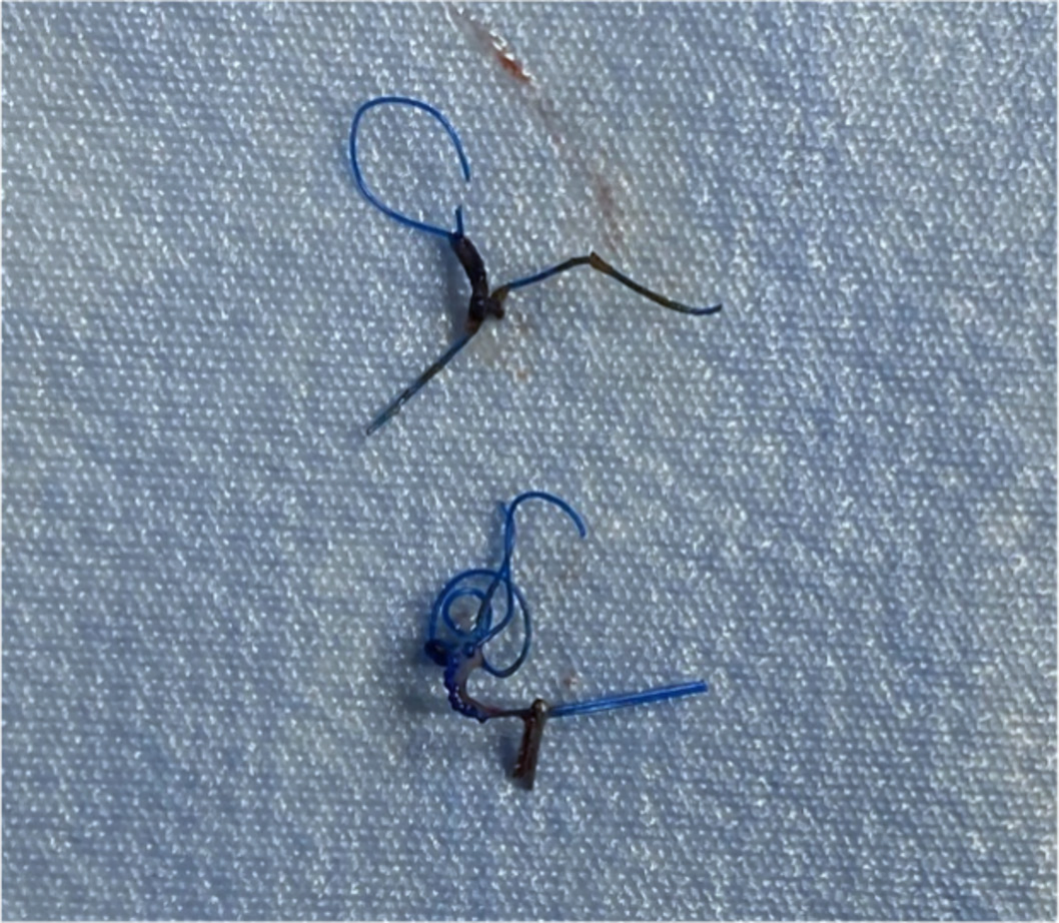
Two endobronchial sutures that were removed from the bronchial tree.

**FIGURE 4:**
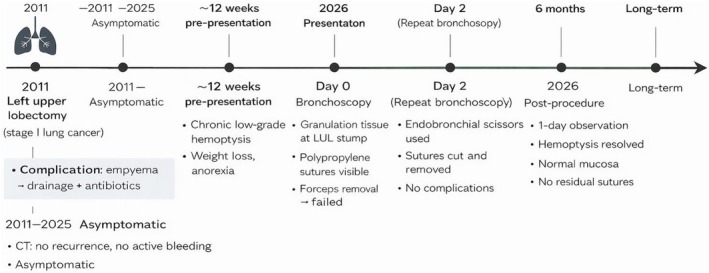
Timeline.

A 54‐year‐old male patient presented to the hospital after 1 week of continuous low‐grade haemoptysis. He had a previous history of left upper lobectomy in 2011 for stage 1 lung cancer. His surgery was complicated by empyema, which was treated with drainage and antibiotics. The patient presented 14 years post his index surgery to the clinic with 12 weeks of chronic low‐grade haemoptysis, weight loss and loss of appetite. CT chest with contrast showed no evidence of active bleeding or recurrence of cancer. The patient was admitted and underwent a bronchoscopy. The bronchoscopy was performed under light sedation anaesthesia using fentanyl and midazolam with cardiopulmonary monitoring in the operating room. The bronchoscopy revealed bleeding from granulation tissue around 2 separate visible polypropylene sutures that were located in the left upper bronchial stump. Attempts to remove the sutures with forceps failed. A repeat bronchoscopy was carried out 2 days later using the same setting, after acquiring flexible endoscopy scissors, used by gastroenterology with a diameter of 2.8 mm that can fit through the working channel of a therapeutic bronchoscope. Once the sutures were identified using the bronchoscope (Figure 1), the scissors were manoeuvred under one loop of the suture and the scissors’ jaws were closed, resulting in cutting of the sutures (Figure 2). They were then grasped by the knots and pulled out gently (Figure 3). No complications including bleeding or airway injury were caused by the procedure. The patient stayed in the hospital for 1 day after the procedure with no recurrence of haemoptysis and was discharged home with follow‐up. Both procedures were performed under sedation without complication. Follow‐up bronchoscopy demonstrated complete mucosal healing and absence of granulation or residual suture material 6 months later. The patient continues to be monitored on an annual basis without recurrence (Figure 4).

## 3 Discussion

Polypropylene sutures are frequently used in thoracic surgery and are valued for their durability and permanence; however, their long‐term presence in the airway can, rarely, cause erosion or migration. If the sutures erode into the airway lumen, they can act as a persistent foreign body leading to granulation tissue formation and local inflammation, resulting in a friable mucosa that is susceptible to recurrent haemoptysis. In our patient, the history of empyema likely contributed further to impaired tissue remodelling around the bronchial stump.

Flexible bronchoscopy using endobronchial scissors offers a minimally invasive therapeutic option for this rare problem. Compared with the more invasive alternatives, it avoids the need for general anaesthesia, provides improved access to distal bronchial segments and minimises mucosal trauma. It can also be repeated safely if needed. In this case, endoscopic suture removal was both effective and well tolerated, supporting the effectiveness of this approach.

Prevention strategies during the index operation are essential to minimising long‐term airway complications. The choice of suture material is fundamental in long‐term outcomes. Although non‐absorbable sutures such as polypropylene and silk are commonly used, growing evidence suggests that absorbable monofilament sutures reduce the risk of late stump complications including granulation tissue and suture migration.

Experimental data has shown that stapled closures result in less granulation formation compared with hand‐sewn sutures in animal models [5], and clinical series have reported fewer late stump issues with absorbable and mechanically applied materials [6, 7]. Reinforcing the bronchial stump with pleura, pericardial fat pad or intercostal muscle may further reduce the risk of delayed complications. Meta‐analyses indicate that coverage techniques significantly decrease bronchopleural fistula rates following major lung resection [8], and narrative reviews support the concept that enhanced vascularity and mechanical protection promote more reliable healing, particularly in high‐risk patients [9]. The way the bronchial stump is closed can have lasting effects on airway integrity. Several studies have shown that stapled closure provides even tension distribution and carries a lower risk of contamination, which may translate into fewer stump‐related complications compared with hand‐sewn techniques [6, 7]. Other observational studies, however, have suggested that the choice of closure method alone does not fully determine fistula risk, especially in patients with significant comorbidity or postoperative infection [10]. These findings highlight that stump integrity is multifactorial and is influenced by patient as well as technical factors.

Finally, careful postoperative surveillance, especially in patients with postoperative complications is crucial. Early proactive bronchoscopic examination allows for direct visualisation of the stump and early detection of any complications, especially in the high‐risk postoperative setting.

In conclusion, endobronchial sutures are a rare cause of late post‐lobectomy haemoptysis and should be considered in the diagnosis. Flexible bronchoscopy with endobronchial scissors is a safe, minimally invasive method for suture removal. Careful surgical technique and appropriate suture selection are key to preventing this complication.

## Author Contributions

Talal AlSaeed drafted the manuscript, performed the literature review and prepared the final written version. Sulaiman Khadadah is the primary clinician for the case; performed the procedure; contributed to case conception and critical revision of the manuscript. Abdullah Alelewah, Hassan Jamaluddin, Essa AlGunaim, Adel Ayed, Derar AlShehab contributed to manuscript review, editing and critical revisions for important intellectual content. All authors approved the final version of the manuscript.

## Ethics Statement

Written informed consent was obtained from the patient for the publication of this case report and accompanying images in accordance with institutional and journal requirements. Formal ethics committee approval was not required for this single‐patient case report.

## Consent

The authors declare that written informed consent was obtained for the publication of this manuscript and accompanying images and attest that the form used to obtain consent from the patient complies with the journal's requirements as outlined in the author guidelines.

## Conflicts of Interest

The authors declare no conflicts of interest.

## Data Availability

The data that support the findings of this study are available from the corresponding author upon reasonable request.

## References

[1] D. O'Gurek and H. Y. J. Choi , “Hemoptysis: Evaluation and Management,” Chest 162, no. 5 (2022): e207–e215. 35166503

[2] H. Ittrich , J. Bickenbach , and H. Klose , “The Diagnosis and Treatment of Hemoptysis,” Deutsches Ärzteblatt International 114, no. 21 (2017): 371–381. 28625277 10.3238/arztebl.2017.0371 PMC5478790

[3] Y. Shiraishi , Y. Nakajima , S. Tanaka , et al., “Late Postoperative Complications After Pulmonary Lobectomy,” Interactive Cardiovascular and Thoracic Surgery 12, no. 3 (2011): 428–432.

[4] D. Shure , “Endobronchial Suture: A Foreign Body Causing Chronic Cough,” Chest 100, no. 5 (1991): 1193–1196. 1935271 10.1378/chest.100.5.1193

[5] Y. Izumi , M. Kawamura , M. Gika , and H. Nomori , “Granulation Tissue Formation at the Bronchial Stump Is Reduced After Stapler Closure in Comparison to Suture Closure in Dogs,” Interactive Cardiovascular and Thoracic Surgery 10, no. 3 (2010): 356–359. 20007204 10.1510/icvts.2009.219006

[6] D. Weissberg and M. Kaufman , “Suture Closure Versus Stapling of the Bronchial Stump in 304 Lung Cancer Operations,” Scandinavian Journal of Thoracic and Cardiovascular Surgery 26, no. 2 (1992): 125–127. 1439642 10.3109/14017439209099066

[7] M. Zakkar , R. Kanagasabay , I. Hunt , et al., “No Evidence That Manual Closure of the Bronchial Stump Has a Lower Failure Rate Than Mechanical Stapler Closure,” Interactive Cardiovascular and Thoracic Surgery 18, no. 4 (2014): 488–493. 24351508 10.1093/icvts/ivt502 PMC3957281

[8] M. Di Maio , F. Perrone , C. Deschamps , et al., “Impact of Bronchial Stump Coverage on the Risk of Bronchopleural Fistula After Pneumonectomy: A Meta‐Analysis,” European Journal of Cardio‐Thoracic Surgery 48 (2015): 196–200. 25342849 10.1093/ejcts/ezu381

[9] T. Karasaki , S. Mihara , and S. Fujimori , “Bronchial Stump Reinforcement Using Adjacent Tissue in High‐Risk Patients,” Current Challenges in Thoracic Surgery 7 (2025): 7.

[10] P. Skrzypczak , M. Roszak , M. Kasprzyk , et al., “Technique of Stump Closure Has no Impact on Post‐Pneumonectomy Bronchopleural Fistula in NSCLC: A Cross‐Sectional Study,” Journal of Thoracic Disease 14, no. 9 (2022): 3343–3351. 36245618 10.21037/jtd-22-240 PMC9562551

